# Seroprevalence of human brucellosis in Morocco and associated risk factors

**DOI:** 10.14202/vetworld.2022.2224-2233

**Published:** 2022-09-17

**Authors:** Kaoutar Faddane, Houda Moumni, Imad Cherkaoui, Mohammed Lakranbi, Salsabil Hamdi, Sayeh Ezzikouri, Rachid Saile, Mohamed El Azhari

**Affiliations:** 1 Laboratory of Bacteriology, Institut Pasteur du Maroc, Casablanca, Morocco; 2 Laboratory of Biology and Health URAC34-Metabolic and Immunologic Pathology Research Team, Faculty of Science, Ben M’sik, Hassan II University, Casablanca, Morocco; 3 Directorate of Epidemiology and Disease Control, Ministry of Health, Rabat, Morocco; 4 Environmental Health Laboratory, Institut Pasteur du Maroc, Casablanca, Morocco; 5 Laboratory of Virology, Institut Pasteur du Maroc, Casablanca, Morocco

**Keywords:** human brucellosis, risk factors, seroprevalence, Sidi Kacem (Morocco)

## Abstract

**Background and Aim:**

Brucellosis is a prevalent infectious zoonotic disease that affects humans, livestock, and wildlife in many parts of the world. A cross-sectional study was conducted to estimate the seroprevalence and risk factors of brucellosis among farmers and patients attending six health centers in Sidi Kacem province (northwestern Morocco).

**Materials and Methods:**

Blood samples (3-5 mL) were collected. Among 1283 participants, 351 were males and 932 were females and tested for Brucella antibodies using rose Bengal plate test and immunoglobulin (Ig)M/IgG enzyme-linked immunosorbent assay (ELISA) for confirmation.

**Results:**

The seroprevalence of brucellosis was 33.20% (426/1283) with a higher risk among males and rural residents. The univariable analysis revealed that contacting cattle, handling abortion products and manure, and consuming undercooked beef and goat meat were all risk factors for brucellosis. Furthermore, raw milk and milk derivatives were risk factors strongly linked to brucellosis.

**Conclusion:**

Our findings indicate a high prevalence of brucellosis associated with the consumption of raw meat, raw dairy products, milk, and close contact with infected animals. However, there are some limitations to this study, such as we did not use the ELISA test on all sera collected and individuals under the age of 18 were not included in the study. Moreover, building a database on the occurrence of brucellosis and associated epidemiological factors is critical for providing informed advice to policymakers to improve control strategies against this disease in Morocco.

## Introduction

The pathogens of brucellosis are small, fastidious, Gram-negative coccobacilli belonging to the genus *Brucella*. *Brucella abortus*, *Brucella melitensis*, and *Brucella suis* infect cattle, small ruminants, and pigs, respectively. Moreover, these species are of particular importance in human and animal infections worldwide. In addition, other species of concern are *Brucella canis*, which infects dogs, and *Brucella ovis*, which infects sheep [1]. Brucellosis is mostly transmitted to humans through direct contact with infected animals, their tissues (such as placenta or aborted tissues), or consumption of their products (such as dairy products) [2]. Person-to-person transmission is rare but can occur through transplacental transmission, breastfeeding, and, rarely, through sexual intercourse, organ transplants, and blood transfusions [3]. In humans, brucellosis usually manifests as a series of nonspecific clinical signs, including malaise, fatigue, arthritis, and fever. Chronicity and recurrent febrile states with joint pain are common sequelae [2].

According to the World Health Organization (WHO), more than 500,000 new cases of human brucellosis are reported each year worldwide. However, it is estimated that the number of undiagnosed cases is 4 times higher [4, 5]. Despite this underestimation, the annual incidence rate of brucellosis in endemic areas worldwide varies widely from <0.01 to more than 200 cases/100,000 population, depending on geographic area, level of hygiene, dietary habits, occupation, and other factors [6]. The highest prevalence was observed in the Mediterranean basin (North Africa, Portugal, Spain, southern France, Italy, Greece, and Turkey), Mexico, South and Central America, Eastern Europe, Asia, Africa, the Caribbean, and the Middle East [7]. In the Maghreb region (Morocco/Algeria/Tunisia), brucellosis remains a major public health problem, despite the various program strategies that have been implemented to control the disease. The presence of human brucellosis in the Maghreb still seems to be underestimated and its epidemiological situation remains largely unknown [8].

To the best of our knowledge, no human brucellosis research has been conducted in Morocco in the last decade. Epidemiological surveillance of animal brucellosis by veterinary authorities has revealed that this disease is enzootic in different regions of the country, with different prevalence in different categories of livestock. According to the survey on animal brucellosis conducted by the “Office National de Sécurité Sanitaire des Produits Alimentaires” in 2010, estimated seroprevalences were 4.92% in livestock and 2.15% in cattle [9].

This study aimed to estimate the prevalence of human brucellosis among farmers and patients attending health centers (HCs) in the province of Sidi Kacem, Morocco. In addition, the study aimed to identify potential risk factors for this disease among patients in the indicated study area. Our survey contributes to the constitution of a database on the occurrence of brucellosis and associated epidemiological factors, to provide an informed recommendation to policymakers to improve brucellosis control strategies in Morocco.

## Materials and Methods

### Ethical approval and Informed consent

Ethical approval was obtained from the “Ethics Committee for Biomedical Research, Rabat, Morocco” before the study (CERB reference number N/R 34/16). Informed consent was obtained from all participants (we recruited only adult participants) by explaining in writing and verbally the purpose of the study. The study was conducted in accordance with the principles of the Helsinki Declaration. Participation in our study was voluntary, anonymous, confidential, and only for research purposes to protect the privacy and ensure data integrity.

### Study period and location

**Figure-1:**
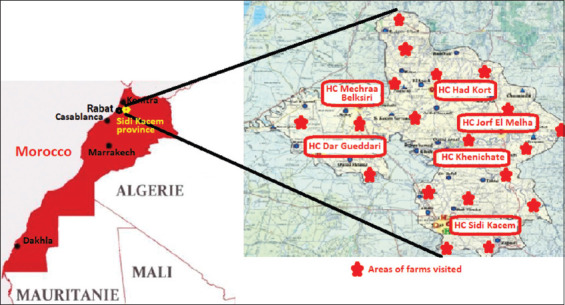
Sampling area sites [Source: Base maps from IECD, France and www.VYMaps.com].

The study was conducted from December 17 to 31, 2017 in six HCs and numerous farms covering the entire province of Sidi Kacem, located in northwestern Morocco and composed of 29 communes. According to the Kingdom of Morocco’s High Commission for Planning (KMHCP) (web), the population of Sidi Kacem province in 2017 was approximately 476,600, the majority of whom lived in rural areas with a population density of 150.6/km². The province had six HCs of very unequal size, including the city of Sidi Kacem (the largest), the other five with a rural context are called Mechraa Belksiri, Jorf El Melha, Dar Gueddari, Had Kort and Khenichate (Figure-1). The sample included adult men and women who agreed to participate after giving their informed consent.

### Study design and sample size determination

A cross-sectional study was conducted to identify the prevalence and risk factors of human brucellosis among farmers and patients attending the six HCs in Sidi Kacem province. In the absence of data on human brucellosis seroprevalence estimates in Morocco, we first considered an overall convenience sample of approximately 1400 individuals in the 6 HCs and farms based on a power of 80%, a type I error of 5%, a nonresponse rate of 10%, and an expected seroprevalence around 12% according to previous studies [10, 11]. Furthermore, using the data from the KMHCP relating to the geographic distribution of Sidi Kacem province residents, the samples were planned as follows: 140 cases in Dar El Geddari, 140 cases in Had Kourt, 160 cases in Mechraa Belksiri, 220 cases in Sidi Kacem, 140 cases in Jorf El Melha, 150 cases in Khenichat and 430 cases in the farms.

### Study participants and data collection

The data collection was conducted by a team of 13 people, including two physicians, two veterinarians, one researcher, seven nurses, and two drivers. A structured questionnaire in French and Arabic was used to interview a total of 1283 people. Sociodemographic characteristics, including gender, age, education level, place of residence (urban/rural), and potential risk factors such as type of contact with animals (cats, sheep, and goats), exposure to aborted fetuses/animal parts, and consumption patterns of dairy products (milk and derivatives), either raw, informal, or pasteurized, were collected. A list of farms was used to select one to three random samples of adult individuals stratified by geographic location. All study subjects were visited by a specialized team and samples were collected on-site using the mobile laboratory of the Pasteur Institute of Morocco. A standard vacutainer tube was used to collect a 3–5 mL venous blood sample from each patient. The sample was kept at room temperature (20–25°C) for 30 min to facilitate coagulation and centrifuged at 1509× *g* for 5 min to obtain the clear serum. All sera were separated into labeled 1.8 mL cryotubes and transported to the Pasteur Institute of Morocco in a cold box and stored at 4°C until use.

### Blood examination for brucellosis

Two tests, agglutination and nonagglutination, were used to confirm the disease, according to CDC and WHO guidelines [5, 12]. The rose Bengal plate test (RBPT) was used to screen sera, and positive samples were then subjected to an indirect enzyme-linked immunosorbent assay (ELISA) (immunoglobulin [Ig] M ELISA and IgG ELISA).

Sera, RBPT, ELISA reagents, and controls were thawed and brought to room temperature before screening for anti-*Brucella* antibodies in the microbiology laboratory of the Pasteur Institute of Morocco. Rose Bengal plate test was performed with a commercial *Brucella* antigen (*B. abortus*, Crescent Diagnostics, KSA) according to the manufacturer’s instructions. Briefly, 30 mL of test serum was mixed with 30 mL of Rose Bengal antigen on a clean glass slide and stirred with a disposable stick. The slide was rotated manually for 5–6 min. The presence of agglutinating clusters indicates a positive reaction, while their absence indicates a negative test. A known positive and negative sera (confirmed as positive or negative in our laboratory and stored at −80°C) were used as controls. The RBPT-positive sera were then subjected to an ELISA as a confirmatory test, while the negative sera were stored at −20°C. *Brucella*-specific IgG and IgM ELISA antibody titers in serum samples were determined as previously described (Vircell *Brucella* IgM ELISA and Vircell *Brucella* IgG ELISA, Spain). Briefly, 96-well microtiter plates were coated with LPS of *B. abortus* strain S-99 diluent and serum samples were added to the wells. The plates were incubated at 37°C for 45 min and washed before the addition of peroxidase-conjugated anti-human IgG or IgM was added to the wells. Plates were incubated at 37°C for 30 min, washed again and tetramethylbenzidine was added. The reaction was stopped by the addition of sulfuric acid after 20 min of incubation in the dark and the plates were read at 450 nm in a spectrophotometer (Institut Pasteur du Maroc, Morocco).

The presence or absence of *Brucella* anti-LPS antibodies was determined by comparing optical densities (OD) to cut-off values obtained from the positive control. Therefore, samples considered positive are those with OD values ≥ positive cut-off values, samples considered negative are those with OD values < positive cut-off values

Moreover, a person was considered seropositive when he/she tested positive for both RBPT and ELISA (IgM and/or IgG).

### Statistical analysis

Categorical variables were presented as numbers and proportions for descriptive statistics. The Chi-square test was used to perform a univariate analysis of categorical variables associated with *Brucella* seropositivity. The association between seropositivity and exposure to risk factors was reported using an odds ratio (OR) with a 95% confidence interval (CI). All statistical analyses were carried out using R software for Windows (https://www.r-project.org) and GraphPad PRISM version 6.0e (GraphPad Software, San Diego, CA, USA). Every statistical test was two-sided. All statistical procedures were performed with R software for Windows and GraphPad PRISM version 6.0e (GraphPad Software). p < 0.05 was considered statistically significant. All statistical tests were two-sided.

## Results

A total of 1283 human blood samples were obtained from HCs, including 146 cases from Mechraa Belksiri, 121 cases from Dar Gueddari, 133 cases from Had Kort, 122 cases from Jorf El Melha, 142 cases from Khenichate, 190 cases from Sidi Kacem, and 430 cases from the farms.

The participants in the current study ranged in age from 18 to 83 years (median 42.79), with 932 women (72.6%), and 351 men (27.4%) taking part. The RBPT found 38.04% (488/1283) seroprevalence for brucellosis, but ELISA found only 87.30% (426/488) positive for IgM and/or IgG. The remaining 12.70% (62/488) were false positives from the RBPT ELISA. The combined seroprevalence (sera positive by both RBPT and ELISA) was 33.20% (426/1283); of these, 212 individuals were farmers (49.77%), and 214 individuals included in the six HCs (50.23%). On the other hand, in the HCs, positive sera were detected in 28.10% (41/146), 9.90% (12/121), 11.28% (15/133), 19.67% (24/122), 29.58% (42/142), and 18.60% (80) of the individuals included in Mechraa Bel Ksiri, Dar Gueddari, Had Kort, Jorf El Melha, Khenichete, and Sidi Kacem, respectively. Positive ELISA tests showed that 394 cases (30.71) were IgG positive, 30 cases (2.39%) were IgM positive, and 2 cases (0.16%) were positive for both IgG and IgM (Table-1).

**Table-1:** Origin and distribution of brucellosis among study respondents (n = 1283).

Origin of tested individuals	Total (%)	Sero negativity n, (%)	RBPT^+ve^/ELISA^+ve^ n, (%)	RBPT^+ve^/ELISA IgG^+ve^; n, (%)	RBPT^+ve^/ELISA IgM^+ve^; n, (%)	RBPT^+ve^/ELISA IgM-IgG^+ve^; n, (%)
HC Mechraa Belksiri	146 (11.38)	104 (12.14)	41 (9.62)	36 (9.14)	5 (16.67)	0
HC Dar Gueddari	121 (9.83)	109 (12.72)	12 (2.82)	10 (2.54)	2 (6.67)	0
HC Had Kort	133 (10.37)	118 (13.77)	15 (3.52)	12 (3.04)	3 (10.00)	0
HC Jorf El Melha	122 (9.51)	98 (11.44)	24 (5.63)	23 (5.84)	1 (3.33)	0
HC Khenichete	142 (11.08)	100 (11.67)	42 (9.86)	38 (9.64)	4 (13.33)	0
HC Sidi Kacem	190 (14.81)	110 (12.84)	80 (18.78)	74 (18.78)	5 (16.67)	1 (50.00)
Farmers	430 (33.82)	218 (25.44)	212 (49.77)	201 (51.02)	10 (33.33)	1 (50.00)
Total	1283	875	426	394	30	2

RBPT=Rose bengal plate test, ELISA=Enzyme-linked immunosorbent assay, HC=Health centers

Table-2 summarizes the analysis of brucellosis seroprevalence according to sociodemographic variables (gender, region of residence, and education level). Univariate logistic regression analysis showed that being male (OR = 1.40, 95%CI: 1.08–1.81, p = 0.0001) and residing in a rural area (OR = 1.88, 95%CI: 1.43–2.48, p = 0.0001) were significantly associated with the occurrence of human brucellosis in individuals. However, the education level of the subjects was not associated with seroprevalence status (p > 0.05).

**Table-2:** Univariate logistic regression of demographic risk factors for brucellosis in Sidi Kacem Province (n = 1283).

Factors	Total	Sero reactivity No. (%)	OR (95% CI)	p-value
Gender*				
Women	932	290 (32.12)	Reference	
Men	351	136 (38.74)	1.40 (1.08–1.81)	0.0001
Education				
Higher education	23	9 (39.13)	Reference	
Secondary school	205	78 (38.04)	1.05 (0.43–2.53)	0.101
Primary school	288	88 (30.55)	1.46 (0.61–3.50)	0.854
Koranic school	73	27 (36.98)	1.09 (0.42–2.87)	0.185
Illiterate	694	224 (32.28)	1.35 (0.57–3.16)	0.690
Region (lived)*				
Urban	366	87 (23.77)	Reference	
Rural	917	339 (36.97)	1.88 (1.43–2.48)	0.0001

OR=Odds ratio, 95% CI=95% Confidence interval. p *<* 0.05 was considered statistically significant.

* Risk Factor

Tables-3–6 present the association between brucellosis and certain risk factors. Contact with livestock, abortion products, and natural manure were important risk factors for brucellosis (Table-3). The subjects in contact with cattle were 2.49 times more likely to be infected with brucellosis than those who had not touched them (p < 0.0001). On the other hand, brucellosis was more frequently detected among subjects who handled abortion products (OR = 1.39, 95% CI: 1.08–1.79, p = 0.009) than those who had not touched them. This is also the case for the subjects who handled the natural manure (OR = 1.63, 95%CI: 1.28–2.08, p < 0.0001) than those who had not touched them.

**Table-3:** Univariate logistic regression of exposure to animals and their products in Sidi Kacem province (n = 1283).

Factors	Total	Sero reactivity No. (%)	OR (95% CI)	p-value
Cattle contact*				
No	526	115 (21.86)	Reference	
Yes	757	311 (41.08)	2.49 (1.94–3.21)	0.0001
Sheep contact				
No	622	203 (32.64)	Reference	
Yes	661	223 (33.74)	1.05 (0.83–1.33)	0.676
Goat contact				
No	1081	351 (32.47)	Reference	
Yes	202	75 (37.13)	1.23 (0.90–1.68)	1.197
Abortion products handling*				
No	898	278 (30.96)	Reference	
Yes	385	148 (38.44)	1.39 (1.08–1.79)	0.009
Natural manure handling*				
No	540	146 (27.04)	Reference	
Yes	743	280 (37.09)	1.63 (1.28–2.08)	<0.0001

OR=Odds ratio, 95% CI=95% Confidence interval. p *<* 0.05 was considered statistically significant,

* Risk Factor

The meat and milk consumption was associated with brucellosis, particularly in beef (p = 0.0005) and goat meat (p = 0.0001), for which the risk was 6.36 and 3.56 times higher, respectively than for those who did not consume them. In particular, when these meats were undercooked (OR = 1.70, 95%CI: 1.14–2.52, p = 0.008) (Table-4). Moreover, consuming cow’s (p < 0.0001) and goat’s (p = 0.032) milk was also associated with brucellosis, with probabilities 1.77 and 1.83 times higher than in non-consumers. Table-5 describes in particular, when drinking farm milk (unpasteurized milk) (OR = 1.97, 95%CI: 1.50–2.60, p < 0.0001), informal milk (OR = 2.09, 95%CI: 1.62–2.69, p < 0.0001), and raw milk (OR = 1.28, 95%CI: 1.02–1.62, p = 0.034) (Table-5).

**Table-4:** Univariate logistic regression of exposure to meat consumption in Sidi Kacem Province (n = 1283).

Factors	Total	Sero reactivity No. (%)	OR (95% CI)	p-value
Consumption of beef meat*				
No	40	3 (7.50)	Reference	
Yes	1248	423 (33.89)	6.36 (1.95–20.76)	0.0005
Consumption of sheep meat				
No	168	54 (32.14)	Reference	
Yes	1115	372 (33.36)	1.06 (0.74–1.49)	0.754
Consumption of goat meat*				
No	848	199 (23.47)	Reference	
Yes	435	227 (52.18)	3.56 (2.78–4.55)	<0.0001
Consumption of well cooked meat				
No	24	6 (25.00)	Reference	
Yes	1259	420 (33.36)	1.50 (0.59–3.81)	0.389
Consumption of undercooked meat*				
No	1173	377 (32.14)	Reference	
Yes	110	49 (44.55)	1.70 (1.14–2.52)	0.008

OR=Odds ratio, 95% CI=95% Confidence interval. p *<* 0.05 was considered statistically significant,

* Risk Factor

**Table-5:** Univariate logistic regression of exposure to milk consumption in Sidi Kacem province (n = 1283).

Factors	Total	Seroreactivity No. (%)	OR (95% CI)	p-value
Consumption of milk				
Cow’s milk*				
No	362	89 (24.59)	Reference	
Yes	921	337 (36.59)	1.77 (1.34–2.33)	<0.0001
Sheep’s milk				
No	1273	420 (32.99)	Reference	
Yes	10	6 (60.00)	3.05 (0.85–10.86)	0.071
Goat’s milk*				
No	1232	402 (32.63)	Reference	
Yes	51	24 (47.06)	1.83 (1.04–3.22)	0.032
Industrial milk				
No	1093	362 (33.12)	Reference	
Yes	190	64 (33.68)	1.02 (0.74–1.42)	0.879
Farm milk*				
No	372	88 (23.66)	Reference	
Yes	891	338 (37.93)	1.97 (1.50–2.60)	<0.0001
Informal milk*				
No	919	261 (28.40)	Reference	
Yes	364	165 (45.33)	2.09 (1.62–2.69)	<0.0001
Raw milk*				
No	635	193 (30.39)	Reference	
Yes	648	233 (35.96)	1.28 (1.02–1.62)	0.034
Boiled milk**				
No	604	275 (45.53)	Reference	
Yes	679	151 (22.24)	0.34 (0.27–0.43)	<0.0001

OR=Odds ratio, 95% CI=95% Confidence interval. p *<* 0.05 was considered statistically significant,

* Risk Factor,

** Protector Factor

Some milk derivatives consumed were also associated with brucellosis, notably cow raib (natural yogurt) made from unpasteurized milk (OR = 1.82, 95%CI: 1.40–2.37, p < 0.0001). In particular, farm raib was 6 times more frequent in subjects who consumed it than in those who did not (OR = 6.12, 95%CI: 2.84–13.19, p < 0.0001) and informal raib (OR = 1.34, 95%CI: 1.03–1.74, p = 0.027). Cow’s butter and goat’s butter were also prevalent for brucellosis: the odds were 1.77 and 4.08 times higher, respectively, for those who consumed cow’s and goat’s milk (p < 0.0001 and p = 0.013, respectively) than for those who did not. Farm butter and informal butter were associated with brucellosis: The odds were 2.09 and 1.83 times higher for those who consumed it (p = 0.0001 and p = 0.007, respectively) than for those who did not. Furthermore, there were no risk factors for brucellosis in subjects consuming other dairy products such as Lben (buttermilk), Jben (Moroccan cheese), ewe’s raib, goat’s raib, industrial butter, and ewe’s butter (Table-6).

**Table-6:** Univariate logistic regression of exposure to milk derivates consumption in Sidi Kacem province (n = 1283).

Factors	Total	Seroreactivity No. (%)	OR (95% CI)	p-value
Consumption of Lben (buttermilk)				
Cow’s Lben**				
No	275	105 (38.18)	Reference	
Yes	1008	321 (31.85)	0.76 (0.57–1.00)	0.048
Sheep’s Lben				
No	1279	424 (33.15)	Reference	
Yes	4	2 (50.00)	2.02 (0.28–14.37)	0.475
Goat’s Lben				
No	1268	422 (33/28)	Reference	
Yes	15	4 (36.36)	0.73 (0.23–2.30)	0.589
Industrial Lben				
No	1029	342 (33.24)	Reference	
Yes	254	84 (33.07)	0.99 (0.74–1.33)	0.960
Farm Lben**				
No	993	366 (36.86)	Reference	
Yes	290	60 (20.69)	0.45 (0.33–0.61)	<0.0001
Informal Lben				
No	76	20 (26.32)	Reference	
Yes	1207	406 (33.64)	1.42 (0.84–2.40)	0.189
Consumption of Raib (natural yoghurt)				
Cow’s Raib*				
No	407	100 (24.75)	Reference	
Yes	876	326 (37.21)	1.82 (1.40–2.37)	<0.0001
Sheep’s Raib				
No	1271	421 (33.12)	Reference	
Yes	12	5 (41.67)	1.44 (0.45–4.57)	0.532
Goat’s Raib				
No	1279	424 (33.15)	Reference	
Yes	4	2 (50.00)	2.02 (0.28–14.37)	0.475
Industrial Raib				
No	1260	417 (33.10)	Reference	
Yes	23	9 (39.13)	1.30 (0.56–3.03)	0.542
Farm Raib*				
No	1248	400 (32.51)	Reference	
Yes	35	26 (25.71)	6.12 (2.84–13.19)	<0.0001
Informal Raib*				
No	947	298 (32.47)	Reference	
Yes	336	128 (38.10)	1.34 (1.03–1.74)	0.027
Consumption of butter				
Cow’s butter*				
No	362	89 (24.59)	Reference	
Yes	921	337 (36.59)	1.77 (1.34–2.33)	<0.0001
Sheep’s butter				
No	1206	396 (32.84)	Reference	
Yes	77	30 (38.96)	1.30 (0.81–2.10)	0.268
Goat’s butter*				
No	1271	418 (32.89)	Reference	
Yes	12	8 (66.67)	4.08 (1.22–13.63)	0.013
Industrial butter				
No	1072	359 (33.49)	Reference	
Yes	211	67 (31.75)	0.92 (0.67–1.27)	0.625
Farm butter*				
No	919	261 (28.40)	Reference	
Yes	364	165 (45.33)	2.09 (1.62–2.69)	<0.0001
Informal butter*				
No	1197	386 (32.25)	Reference	
Yes	86	40 (46.51)	1.83 (1.17–2.84)	0.007
Consumption of Jben (cheese)				
Industrial Jben**				
No	604	275 (45.53)	Reference	
Yes	679	151 (22.24)	0.34 (0.27–0.43)	<0.0001
Farm Jben				
No	1102	370 (33.56)	Reference	
Yes	181	56 (30.94)	0.89 (0.63–1.24)	0.485
Informal Jben				
No	1206	401 (33.25)	Reference	
Yes	77	25 (32.47)	0.96 (0.59–1.58)	0.887

OR=Odds ratio, 95% CI=95% Confidence interval. p *<* 0.05 was considered statistically significant,

* Risk Factor,

** Protector Factor

## Discussion

Brucellosis is a febrile zoonotic disease that presents a severe hazard to humans and domestic animals, which requires a One Health approach characterized by the integration of human and animal health, plants, and ecosystems and encourages joining local, national, and global multidisciplinary efforts to achieve optimal levels of health and collaboration among different disciplines to address complex health problems. The current study is the first comprehensive human study to assess the extent and risk factors for brucellosis transmission in Morocco. Furthermore, no studies on the prevalence of human brucellosis and associated risk factors have been conducted to the best of our knowledge. All samples (1283) were first screened for anti-*Brucella* antibodies using RBPT, and samples that tested positive were confirmed as having *Brucella* antibodies using IgM and IgG ELISA. The overall seroprevalence was 33.20% (426/1283), of which the prevalence of IgG antibodies was 92.49% (394/426), IgM was 7.04% (30/426), and both IgG and IgM were 0.47% (2/426), suggesting that a very large proportion of the population in this region was already infected with *Brucella*. Furthermore, compared to government reports of brucellosis cases from Morocco, which reported 0–27 cases per year (with a median of 2.5 cases/year) in 1999, Algeria with 244.3 cases per million population (MP) in 2017, and Tunisia with 43.5/MP in 2016 [8, 13], and we can observe that the prevalence value found herein is high.

Further, compared to other reports, our result (33.20%) is higher than rates ranging from 2.6% to 27.1% in Saudi Arabia [14], close to the prevalence reported in Ethiopia (29.4% and 34.1%) [15], but remains lower than 40% among pastoralists in Libya [16] and 63.6% among butcher workers in Nigeria [17].

Moreover, our study identified gender and area of residence as risk factors for *Brucella* seropositivity in men. Men and rural residents who lived in Sidi Kacem province were more likely to be seropositive for brucellosis compared with women and urban residents. The studies in Saudi Arabia, Libya, and Tunisia demonstrated similar results [14, 16, 18]. The higher rate of brucellosis seroprevalence in men is probably due to their greater involvement in feeding animals, raising domestic animals and handling their products, managing vulnerable animals (calves, small ruminants, sick, injured, or pregnant animals), cleaning barns, transporting farm manure, and selling animals, milk, and its by-products (such as cheese, butter, and buttermilk), which puts them at greater risk of infection. In contrast, women are mostly occupied with household chores, parenting, and spend less time with the animals. However, brucellosis is likely to be transmitted to both sexes in the rural area of Sidi Kacem through domestic animals and the consumption of unpasteurized artisanal dairy products; hence, rural people are more vulnerable to brucellosis than urban people. Likely, rural families do not receive the health and sanitation education required to prevent brucellosis transmission to humans.

However, there was no correlation between education level and brucellosis seropositivity in our study. This is in agreement with the previous study [19]. In contrast, Alhoshani *et al*. 2016 [20] reported that the least educated individuals in Saudi Arabia had a higher prevalence rate than individuals with higher education; in Yemen, the socioeconomic and educational factors were independent risk factors for brucellosis [21]. Our result suggests that the habit of consuming meat, milk, and its products is an acquired taste in all socio-educational groups in the province of Sidi Kacem. The consumption habits are similar for all levels of education and could be the reason for our findings. Moreover, contact with livestock is a significant risk factor in our study group. This result is in agreement with others in Africa [4, 22, 23], indicating a potential role for this animal in the epidemiology of brucellosis in Sidi Kacem province. In addition, studies in Egypt, Iran, Saudi Arabia, and Tanzania found that the highest brucellosis prevalences were associated with goats, sheep, and goats, and small and large ruminant contact, respectively [19, 22, 24, 25].

Interestingly in our study, contact with sheep and consumption of their products were not risk factors for brucellosis. However, more investigation is required to confirm that these factors are not associated with the transmission of brucellosis to humans. This is probably due to the low susceptibility of these animals to *B. melitensis*, compared to goats [26]. Moreover, it could also reflect the special and extensive surveillance of sheep by the Moroccan authorities, especially before the period preceding the sacred feast of each year (Eid-Al-Adha = ritual slaughter in the Muslim world) which obliges each family to slaughter a sheep. Our result could be reinforced by a recent study conducted by Azami *et al*. [27] on ruminants in the province of Sidi Kacem. Further, the authors reported that only two sheep from two flocks showed positive brucellosis, and these sheep were able to develop antibodies but cleared the infection.

In addition, regarding the lack of association between brucellosis and contact with goats, we believe that, unlike cattle and sheep, goats, which are very intelligent, agile, and independent animals, generally avoid contact with humans and try to escape if captured.

In the present work, the handling of abortion products and manure are risk factors for brucellosis. The WHO report confirms these findings, revealing that contact with infected materials such as an aborted fetus, placenta, urine, manure, and carcass causes human brucellosis in 60–70% of cases [5]. This is especially true given two factors that contribute to human contamination, that is, the relatively long survival of *Brucella* in the environment (between 21 and 81 days) [28] and transmission through skin contact or inhalation [5]. Furthermore, during the birth of ruminants in Tunisia and several rural areas of Morocco, including Sidi Kacem, almost the entire family participates by removing the offspring [18]. In the absence of personal protective equipment and vaccination against brucellosis, this dangerous intervention, along with manure handling, could pose risk factors for several types of infection, including brucellosis. Moreover, our findings show that consumption of undercooked meat was associated development of human brucellosis. This result, which is consistent with findings from studies conducted in Nigeria and India, indicates that eating undercooked meat is associated with the acquisition of human brucellosis [29, 30]. Indeed, in addition to Moroccans’ consumption of well-cooked meat (e.g., the cooked meat of Moroccan Tajine and Couscous), including in Sidi Kacem, other meals based on undercooked meat dishes are widely consumed and can pose risk factors for several diseases, including brucellosis. In the province of Sidi Kacem, as well as throughout Morocco, Kefta (ground lamb or beef), Kotban (skewers of meat, liver, heart, kidney, spleen of ruminants: Beef, lamb, and goat), and Cutlettes (chops of beef, lamb, and goat) are popular street foods throughout the year and a favorite for grilling at home or outside (restaurants, popular souks, and street vendors), especially at the time of Eid-Al-Adha, when many families prepare Kotban and Cutlettes using the meat of the sacrificed animal. These foods are almost partially cooked because they must be colored but still juicy according to Moroccan standards.

Moreover, despite Moroccan legislation requiring the pasteurization of raw milk, informal dairy chains continue to represent a risk of brucellosis transmission. In some Moroccan cities, these informal channels can market 30% of the milk consumed [31].

The consumption of unpasteurized raw milk and dairy products may pose a risk of developing brucellosis in Sidi Kacem residents. This finding is consistent with other studies from underdeveloped regions such as Ethiopia, Iran, Saudi Arabia, and Tunisia [15, 19, 20, 32], but it contrasts with findings from developed countries where all dairy products are pasteurized [33, 34]. The purchase of unpasteurized artisanal dairy products (milk, butter, raib, Jben, Lben, etc.) from local farms, informal dairy chains, or street vendors (i.e., of unknown source, possibly milk from infected animals) is widespread in Sidi Kacem province as in other regions of Morocco. Furthermore, traditional consumption patterns for these products are especially prevalent, and some consumers prefer traditional milk preparations. Our survey confirms these findings, as the majority of people tested for brucellosis reported purchasing and consuming unpasteurized milk and its derivatives, believing that boiling milk alters its taste and suppresses or diminishes its qualities.

While the consumption of Jben (also called Jben Beldi = soft cheese) made from unpasteurized milk has been reported in some countries (including Morocco) as an important risk factor for brucellosis [13, 35, 36], we found no association between its consumption and this disease. Our findings agree with those of a study conducted in Yemen, where soft cheese is typically made from cow’s milk, a source with a very low brucellosis prevalence [21]. The absence of *Brucella* in Sidi Kacem, as well as elsewhere in Morocco, could be attributed to a change in the method of preparation. In this regard, and in contrast to the past, when it took 1–10 days to produce Jben from natural milk without any chemical additives, most women now prefer a simpler and faster process that takes <12 h. They simmer the milk before adding buttermilk (Lben), vinegar, herbs, and spices to taste. Other women heat the whole milk and add artichoke chokes, also known in French as barbe d’artichaut, foin d’artichaut, chardonette, and nyaq or hakka in Moroccan Arabic. We believe that the addition of salts, buttermilk, herbs, aromatics, vinegar, and/or artichoke chokes to heated whole milk is a lethal factor for *Brucella*. More research is needed to determine whether the Moroccan method of rapid cheese preparation results in *Brucella*-free cheese.

We also did not find an association between Lben (buttermilk) consumption and brucellosis. This lack of association can be explained by the increased fermentation of cow, sheep, and goat milk, which is usually carried by the spontaneous enzymatic activities of lactic acid bacteria, which are known to inhibit the growth of several pathogenic bacteria, primarily due to the reduction of pH [37]. In our study, however, consumption of unpasteurized butter (from cows and goats) was a significant source of infection. This result is consistent with previous reports [19, 32].

Finally, it is interesting to note that we observed a protective effect of boiled milk (OR = 0.34, 95%CI: 0.27–0.43, p < 0.0001), cow Lben (OR = 0.76, 95%CI: 0.57–1.00, p = 0.048), farm Lben (OR = 0.45, 95%CI: 0.33–0.61, p < 0.0001), and industrial Jben (OR = 0.34, 95%CI: 0.27–0.43, p < 0.0001). This could be due to the direct effect of improved nutrition and health in those who consume more boiled milk, industrial (pasteurized) Jben, cow, and farm Lben or reflect the effects of unobserved factors that are related to consumption of these foods and brucellosis risk.

There are some limitations to this study. First, instead of using serological tests (RBPT and ELISA) in combination to minimize the measurement of false positive errors, only RBPT-positive sera were subjected to ELISA as a confirmatory test. The second limitation is that the study excluded individuals under the age of 18, who represent a very large sample of the population living in the province of Sidi Kacem. In the future, we hope to expand our study to include all ages as well as domestic animals such as sheep, goats, and cattle by combining serological tests (RBPT and ELISA). This future work will provide us with a global perspective not only on brucellosis seroprevalence in humans and animals but also on the spread of the disease.

## Conclusion

The prevalence of human brucellosis in this study area suggests that human brucellosis is an important public health problem in Morocco. This significant finding could be attributed to three factors, that is, consumption of undercooked meat, traditional use of raw dairy products, and close contact with infected animals, especially cattle. There is a dire need to increase awareness of the disease and its risk factors among community members and health professionals, which could be done through collaboration between public health and veterinary authorities. Moreover, a One Health approach should be strengthened to ensure successful and sustainable brucellosis prevention and control in Morocco.

## Authors’ Contributions

ME, IC, and RS: Conceptualization. ME, RS, KF, IC, ML, and HM: Methodology. SE and HM: Software. ME, RS, and IC: Validation. HM, ML, and SE: Formal analysis. ME, KF, FB, ML, and HM: Investigation. ME and HM: Resources. ME and KF: Data curation. KF, HM, and ME: Drafted the manuscript. RS, IC, and SE: Reviewed and edited the manuscript. SH and HM: Visualization. RS and ME: Supervision. ME: Project administration. All authors have read and approved the final manuscript.
